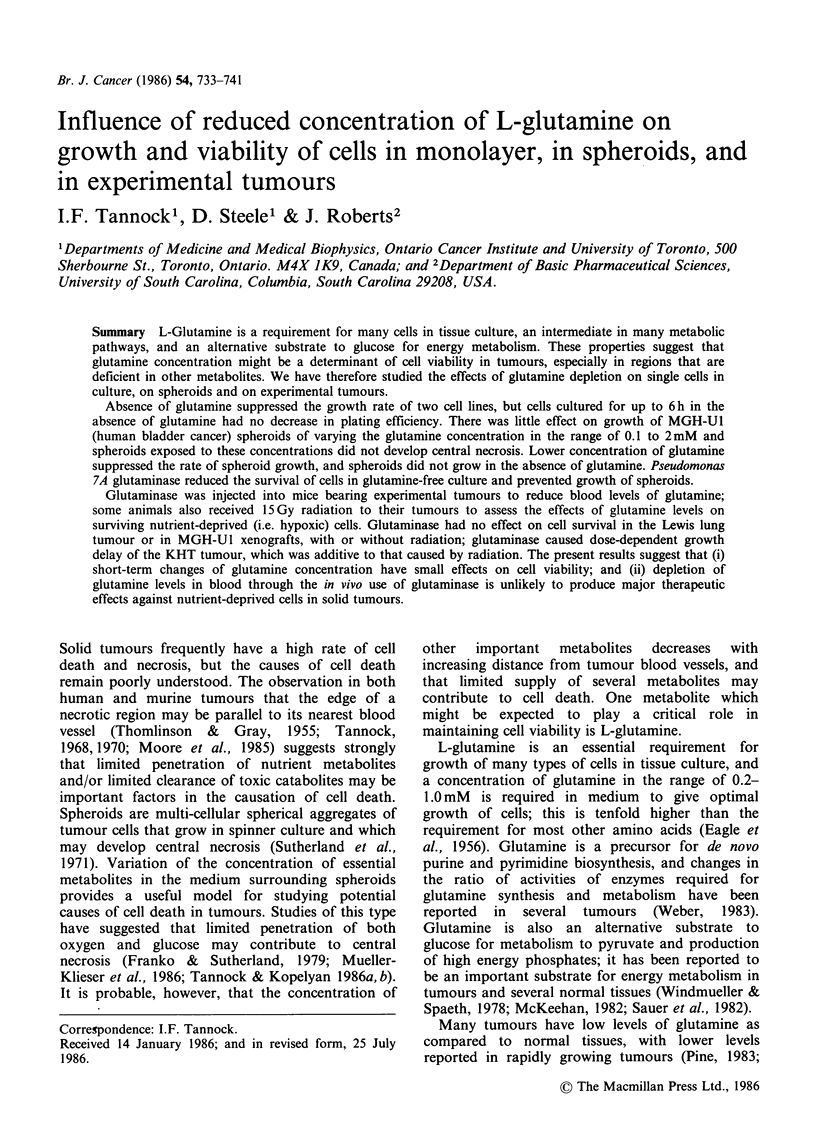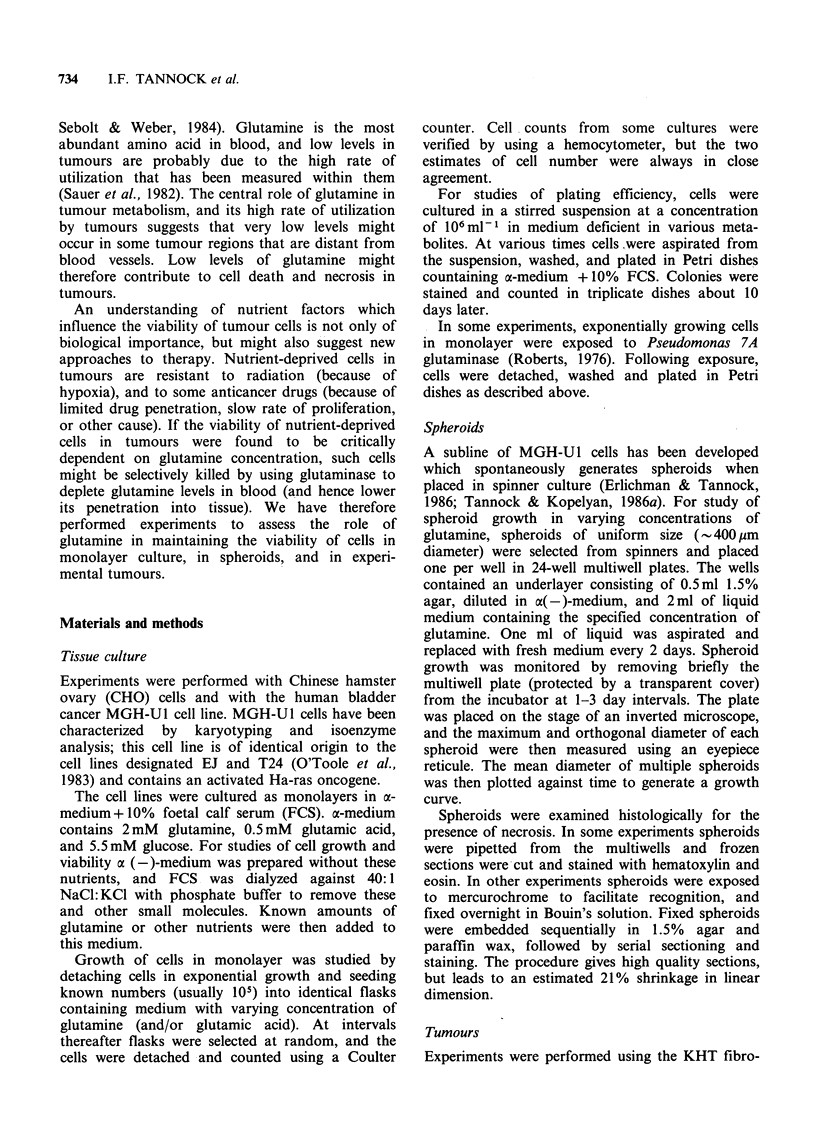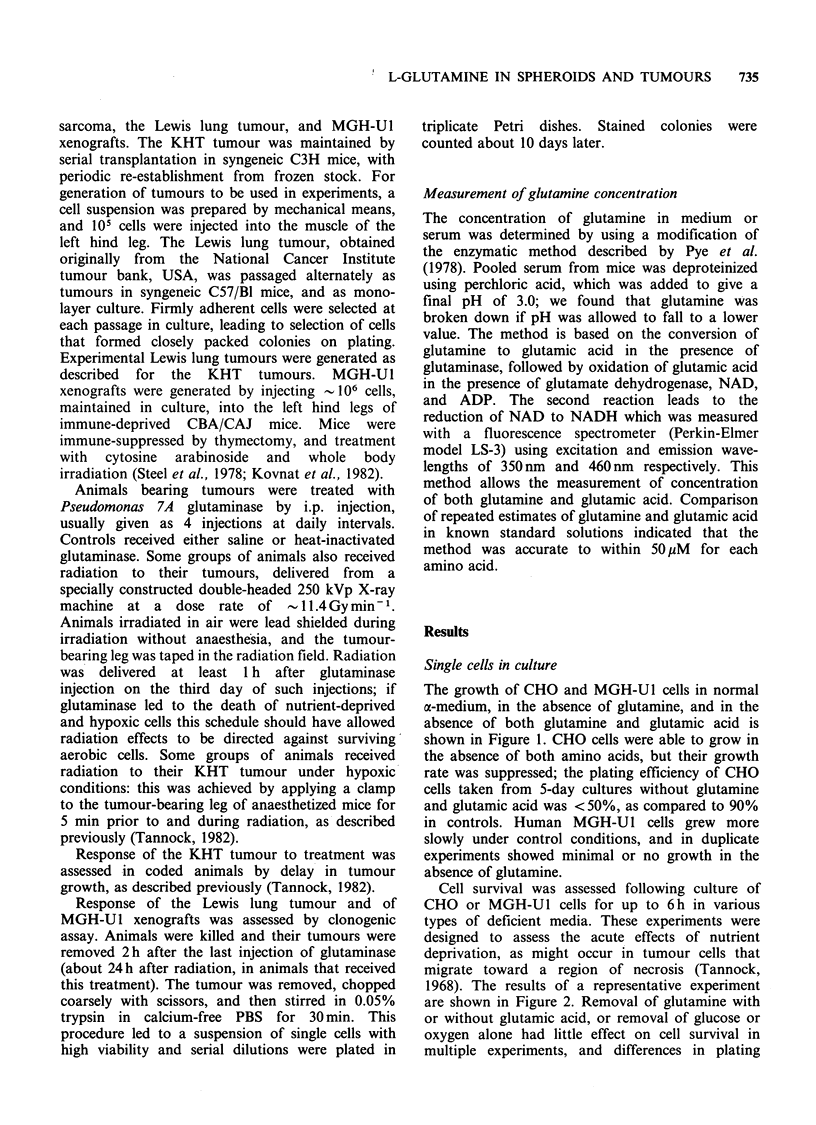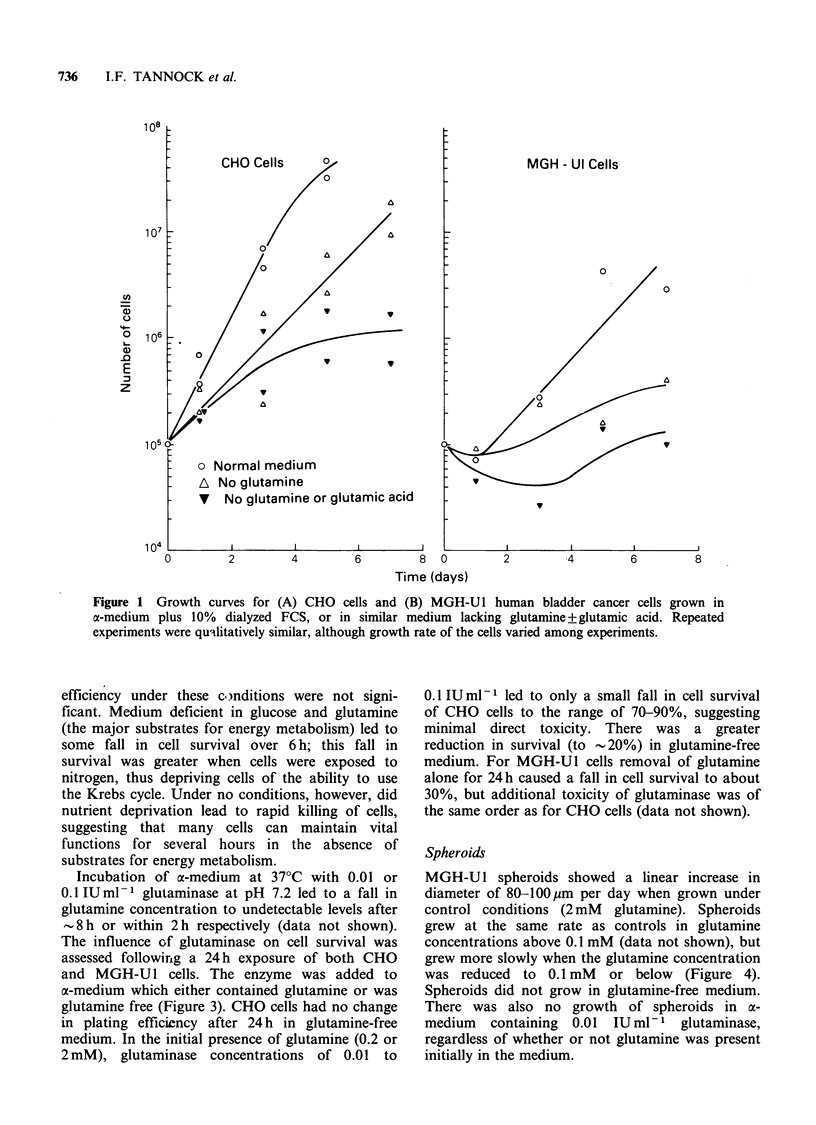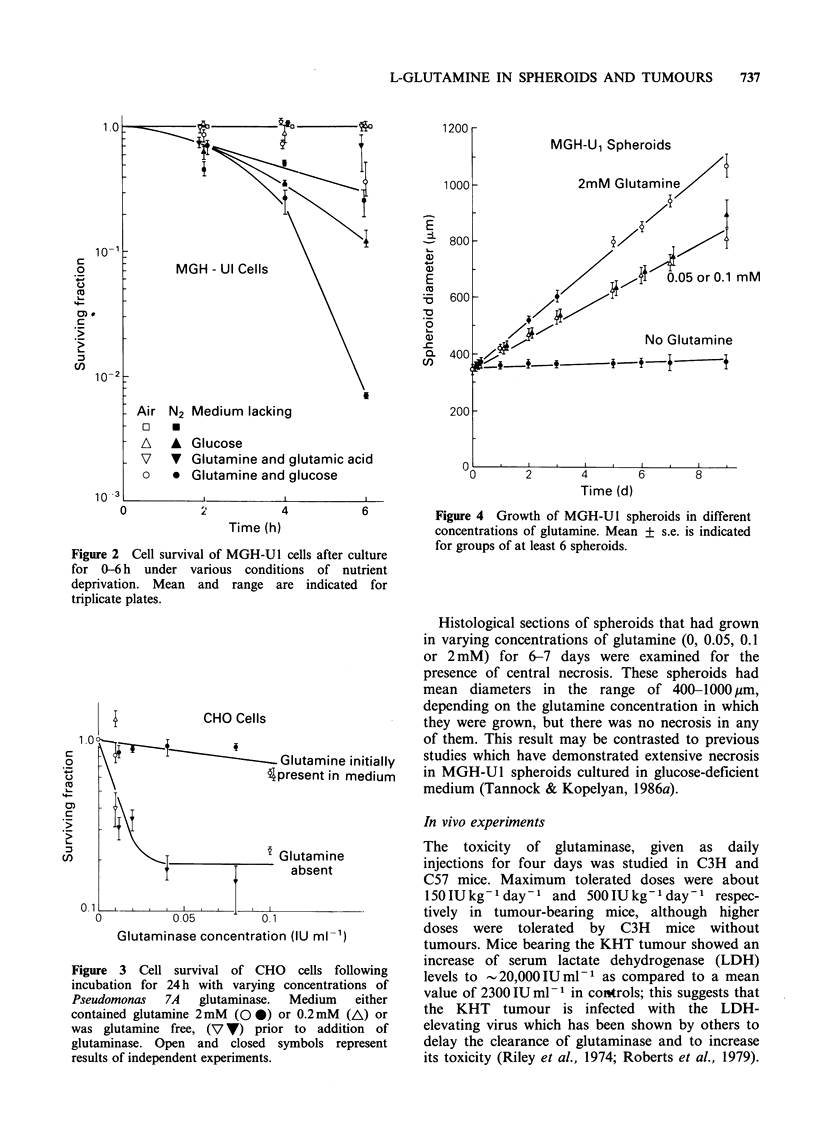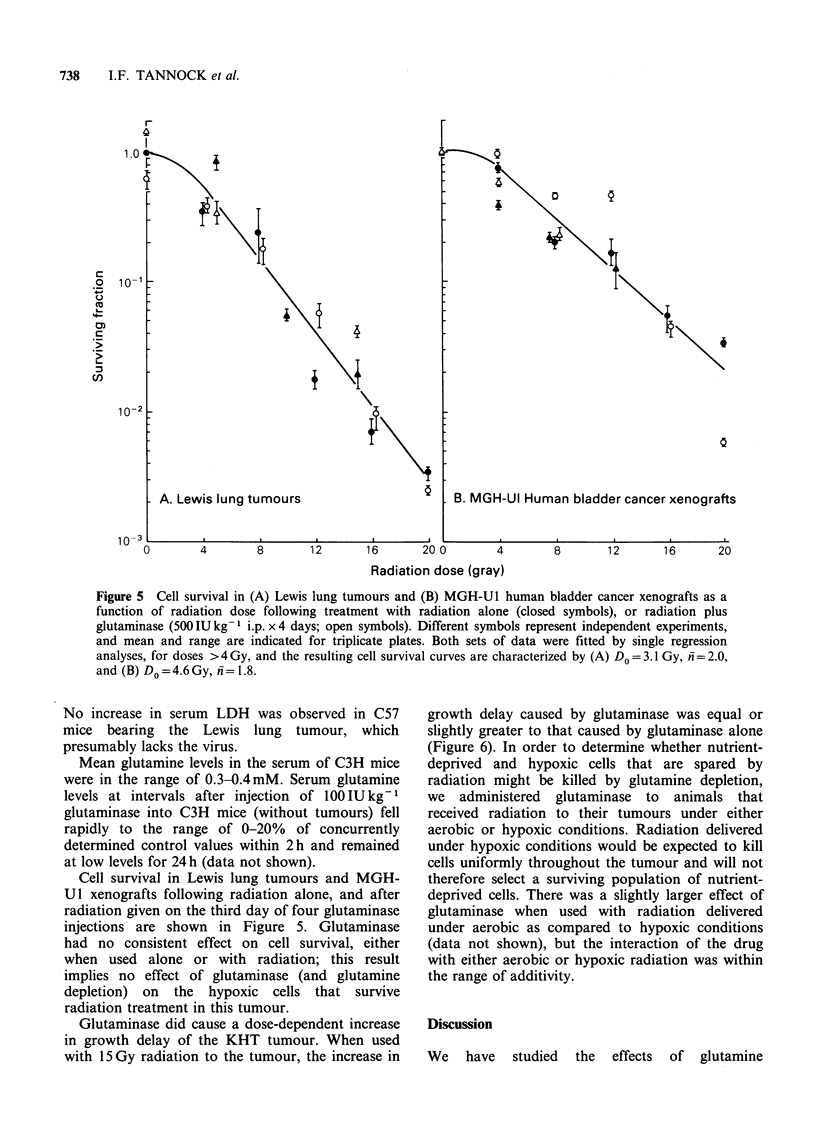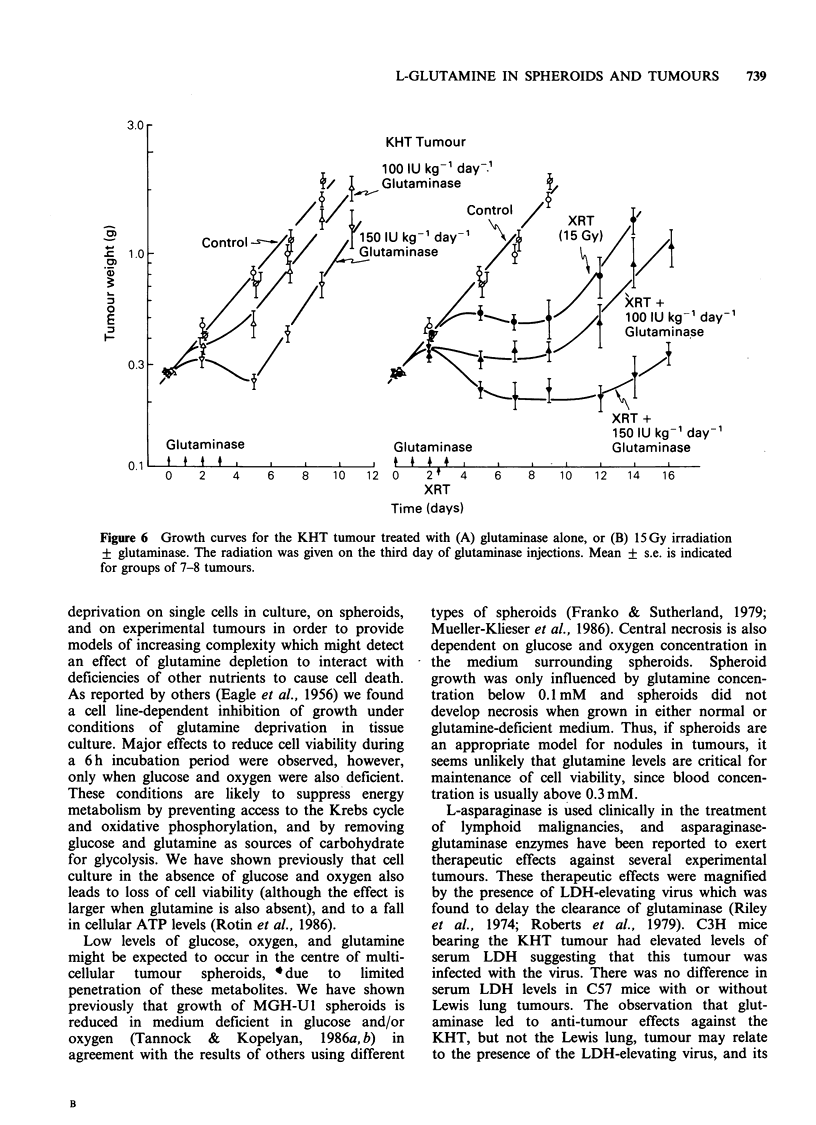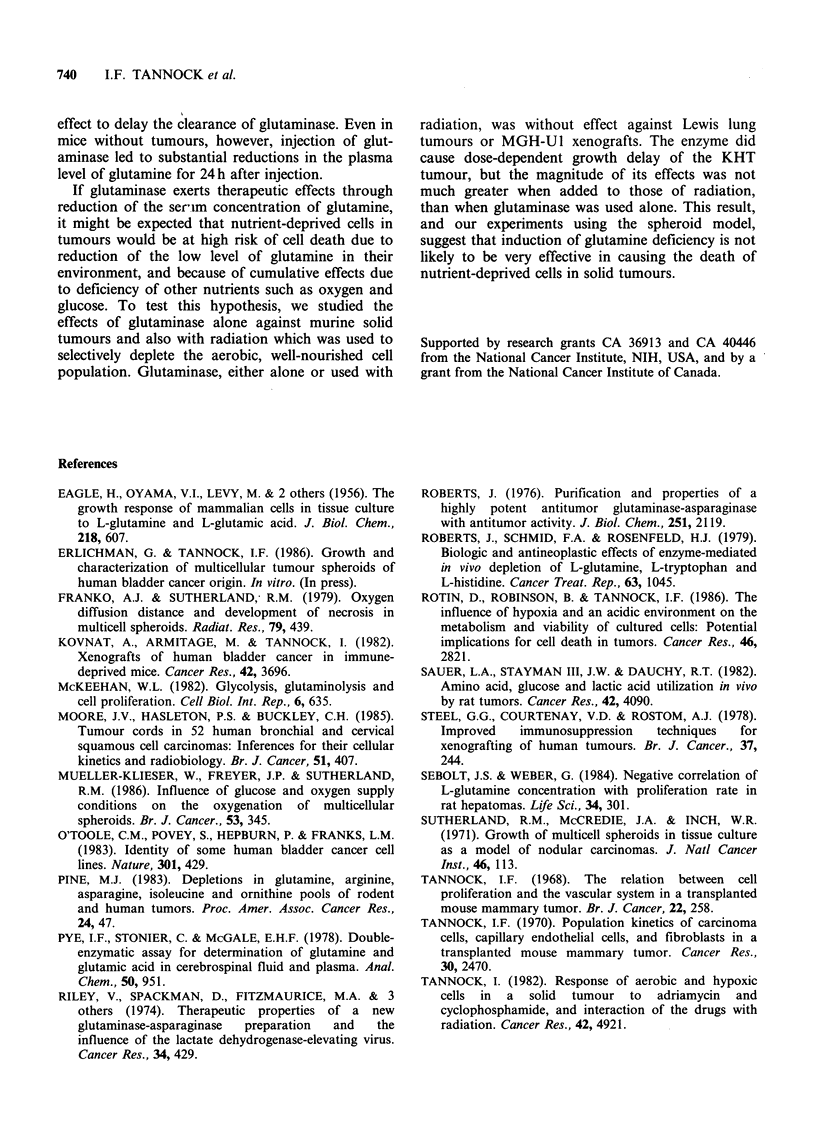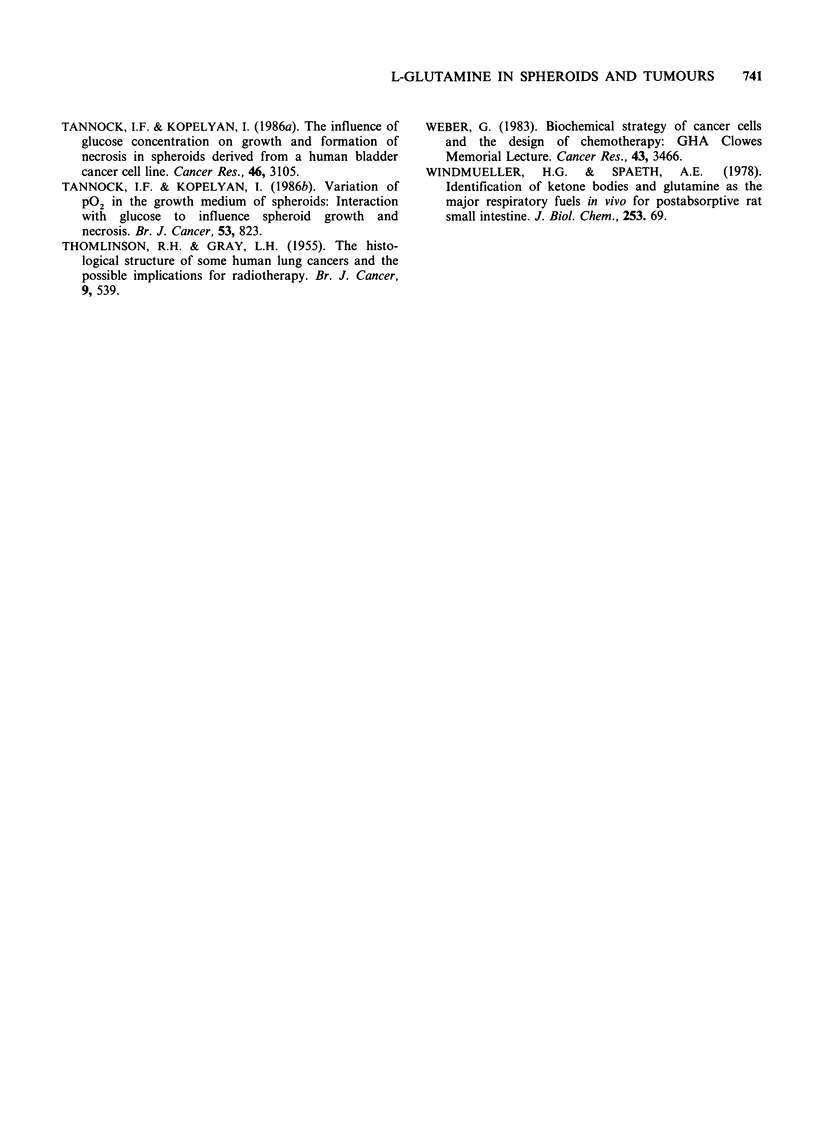# Influence of reduced concentration of L-glutamine on growth and viability of cells in monolayer, in spheroids, and in experimental tumours.

**DOI:** 10.1038/bjc.1986.234

**Published:** 1986-11

**Authors:** I. F. Tannock, D. Steele, J. Roberts

## Abstract

L-Glutamine is a requirement for many cells in tissue culture, an intermediate in many metabolic pathways, and an alternative substrate to glucose for energy metabolism. These properties suggest that glutamine concentration might be a determinant of cell viability in tumours, especially in regions that are deficient in other metabolites. We have therefore studied the effects of glutamine depletion on single cells in culture, on spheroids and on experimental tumours. Absence of glutamine suppressed the growth rate of two cell lines, but cells cultured for up to 6 h in the absence of glutamine had no decrease in plating efficiency. There was little effect on growth of MGH-U1 (human bladder cancer) spheroids of varying the glutamine concentration in the range of 0.1 to 2 mM and spheroids exposed to these concentrations did not develop central necrosis. Lower concentration of glutamine suppressed the rate of spheroid growth, and spheroids did not grow in the absence of glutamine. Pseudomonas 7A glutaminase reduced the survival of cells in glutamine-free culture and prevented growth of spheroids. Glutaminase was injected into mice bearing experimental tumours to reduce blood levels of glutamine; some animals also received 15 Gy radiation to their tumours to assess the effects of glutamine levels on surviving nutrient-deprived (i.e. hypoxic) cells. Glutaminase had no effect on cell survival in the Lewis lung tumour or in MGH-U1 xenografts, with or without radiation; glutaminase caused dose-dependent growth delay of the KHT tumour, which was additive to that caused by radiation. The present results suggest that (i) short-term changes of glutamine concentration have small effects on cell viability; and (ii) depletion of glutamine levels in blood through the in vivo use of glutaminase is unlikely to produce major therapeutic effects against nutrient-deprived cells in solid tumours.


*Br. J. Cancer* (1986) **54**, 733–741

### Influence of reduced concentration of L-glutamine on growth and viability of cells in monolayer, in spheroids, and in experimental tumours

I.F. Tannock[1], D. Steele[1] & J. Roberts[2]

[1]*Departments of Medicine and Medical Biophysics, Ontario Cancer Institute and University of Toronto, 500 Sherbourne St., Toronto, Ontario. M4X 1K9, Canada; and* [2]*Department of Basic Pharmaceutical Sciences, University of South Carolina, Columbia, South Carolina 29208, USA.*

**Summary** L-Glutamine is a requirement for many cells in tissue culture, an intermediate in many metabolic pathways, and an alternative substrate to glucose for energy metabolism. These properties suggest that glutamine concentration might be a determinant of cell viability in tumours, especially in regions that are deficient in other metabolites. We have therefore studied the effects of glutamine depletion on single cells in culture, on spheroids and on experimental tumours.

Absence of glutamine suppressed the growth rate of two cell lines, but cells cultured for up to 6 h in the absence of glutamine had no decrease in plating efficiency. There was little effect on growth of MGH-U1 (human bladder cancer) spheroids of varying the glutamine concentration in the range of 0.1 to 2 mM and spheroids exposed to these concentrations did not develop central necrosis. Lower concentration of glutamine suppressed the rate of spheroid growth, and spheroids did not grow in the absence of glutamine. *Pseudomonas 7A* glutaminase reduced the survival of cells in glutamine-free culture and prevented growth of spheroids.

Glutaminase was injected into mice bearing experimental tumours to reduce blood levels of glutamine; some animals also received 15 Gy radiation to their tumours to assess the effects of glutamine levels on surviving nutrient-deprived (i.e. hypoxic) cells. Glutaminase had no effect on cell survival in the Lewis lung tumour or in MGH-U1 xenografts, with or without radiation; glutaminase caused dose-dependent growth delay of the KHT tumour, which was additive to that caused by radiation. The present results suggest that (i) short-term changes of glutamine concentration have small effects on cell viability; and (ii) depletion of glutamine levels in blood through the *in vivo* use of glutaminase is unlikely to produce major therapeutic effects against nutrient-deprived cells in solid tumours.

Solid tumours frequently have a high rate of cell death and necrosis, but the causes of cell death remain poorly understood. The observation in both human and murine tumours that the edge of a necrotic region may be parallel to its nearest blood vessel (Thomlinson & Gray, 1955; Tannock, 1968, 1970; Moore *et al.*, 1985) suggests strongly that limited penetration of nutrient metabolites and/or limited clearance of toxic catabolites may be important factors in the causation of cell death. Spheroids are multi-cellular spherical aggregates of tumour cells that grow in spinner culture and which may develop central necrosis (Sutherland *et al.*, 1971). Variation of the concentration of essential metabolites in the medium surrounding spheroids provides a useful model for studying potential causes of cell death in tumours. Studies of this type have suggested that limited penetration of both oxygen and glucose may contribute to central necrosis (Franko & Sutherland, 1979; Mueller-Klieser *et al.*, 1986; Tannock & Kopelyan 1986*a*, *b*). It is probable, however, that the concentration of other important metabolites decreases with increasing distance from tumour blood vessels, and that limited supply of several metabolites may contribute to cell death. One metabolite which might be expected to play a critical role in maintaining cell viability is L-glutamine.

L-glutamine is an essential requirement for growth of many types of cells in tissue culture, and a concentration of glutamine in the range of 0.2–1.0 mM is required in medium to give optimal growth of cells; this is tenfold higher than the requirement for most other amino acids (Eagle *et al.*, 1956). Glutamine is a precursor for *de novo* purine and pyrimidine biosynthesis, and changes in the ratio of activities of enzymes required for glutamine synthesis and metabolism have been reported in several tumours (Weber, 1983). Glutamine is also an alternative substrate to glucose for metabolism to pyruvate and production of high energy phosphates; it has been reported to be an important substrate for energy metabolism in tumours and several normal tissues (Windmueller & Spaeth, 1978; McKeehan, 1982; Sauer *et al.*, 1982).

Many tumours have low levels of glutamine as compared to normal tissues, with lower levels reported in rapidly growing tumours (Pine, 1983;

Correspondence: I.F. Tannock.
Received 14 January 1986; and in revised form, 25 July 1986.

© The Macmillan Press Ltd., 1986

Sebolt & Weber, 1984). Glutamine is the most abundant amino acid in blood, and low levels in tumours are probably due to the high rate of utilization that has been measured within them (Sauer *et al.*, 1982). The central role of glutamine in tumour metabolism, and its high rate of utilization by tumours suggests that very low levels might occur in some tumour regions that are distant from blood vessels. Low levels of glutamine might therefore contribute to cell death and necrosis in tumours.

An understanding of nutrient factors which influence the viability of tumour cells is not only of biological importance, but might also suggest new approaches to therapy. Nutrient-deprived cells in tumours are resistant to radiation (because of hypoxia), and to some anticancer drugs (because of limited drug penetration, slow rate of proliferation, or other cause). If the viability of nutrient-deprived cells in tumours were found to be critically dependent on glutamine concentration, such cells might be selectively killed by using glutaminase to deplete glutamine levels in blood (and hence lower its penetration into tissue). We have therefore performed experiments to assess the role of glutamine in maintaining the viability of cells in monolayer culture, in spheroids, and in experimental tumours.

### Materials and methods

### *Tissue culture*

Experiments were performed with Chinese hamster ovary (CHO) cells and with the human bladder cancer MGH-U1 cell line. MGH-U1 cells have been characterized by karyotyping and isoenzyme analysis; this cell line is of identical origin to the cell lines designated EJ and T24 (O'Toole *et al.*, 1983) and contains an activated Ha-ras oncogene.

The cell lines were cultured as monolayers in α-medium + 10% foetal calf serum (FCS). α-medium contains 2 mM glutamine, 0.5 mM glutamic acid, and 5.5 mM glucose. For studies of cell growth and viability α (−)-medium was prepared without these nutrients, and FCS was dialyzed against 40:1 NaCl:KCl with phosphate buffer to remove these and other small molecules. Known amounts of glutamine or other nutrients were then added to this medium.

Growth of cells in monolayer was studied by detaching cells in exponential growth and seeding known numbers (usually $10^5$) into identical flasks containing medium with varying concentration of glutamine (and/or glutamic acid). At intervals thereafter flasks were selected at random, and the cells were detached and counted using a Coulter counter. Cell counts from some cultures were verified by using a hemocytometer, but the two estimates of cell number were always in close agreement.

For studies of plating efficiency, cells were cultured in a stirred suspension at a concentration of $10^6\,ml^{-1}$ in medium deficient in various metabolites. At various times cells were aspirated from the suspension, washed, and plated in Petri dishes countaining α-medium + 10% FCS. Colonies were stained and counted in triplicate dishes about 10 days later.

In some experiments, exponentially growing cells in monolayer were exposed to *Pseudomonas 7A* glutaminase (Roberts, 1976). Following exposure, cells were detached, washed and plated in Petri dishes as described above.

### *Spheroids*

A subline of MGH-U1 cells has been developed which spontaneously generates spheroids when placed in spinner culture (Erlichman & Tannock, 1986; Tannock & Kopelyan, 1986*a*). For study of spheroid growth in varying concentrations of glutamine, spheroids of uniform size (~400 μm diameter) were selected from spinners and placed one per well in 24-well multiwell plates. The wells contained an underlayer consisting of 0.5 ml 1.5% agar, diluted in α(−)-medium, and 2 ml of liquid medium containing the specified concentration of glutamine. One ml of liquid was aspirated and replaced with fresh medium every 2 days. Spheroid growth was monitored by removing briefly the multiwell plate (protected by a transparent cover) from the incubator at 1–3 day intervals. The plate was placed on the stage of an inverted microscope, and the maximum and orthogonal diameter of each spheroid were then measured using an eyepiece reticule. The mean diameter of multiple spheroids was then plotted against time to generate a growth curve.

Spheroids were examined histologically for the presence of necrosis. In some experiments spheroids were pipetted from the multiwells and frozen sections were cut and stained with hematoxylin and eosin. In other experiments spheroids were exposed to mercurochrome to facilitate recognition, and fixed overnight in Bouin's solution. Fixed spheroids were embedded sequentially in 1.5% agar and paraffin wax, followed by serial sectioning and staining. The procedure gives high quality sections, but leads to an estimated 21% shrinkage in linear dimension.

### *Tumours*

Experiments were performed using the KHT fibrosarcoma, the Lewis lung tumour, and MGH-U1 xenografts. The KHT tumour was maintained by serial transplantation in syngeneic C3H mice, with periodic re-establishment from frozen stock. For generation of tumours to be used in experiments, a cell suspension was prepared by mechanical means, and $10^5$ cells were injected into the muscle of the left hind leg. The Lewis lung tumour, obtained originally from the National Cancer Institute tumour bank, USA, was passaged alternately as tumours in syngeneic C57/Bl mice, and as monolayer culture. Firmly adherent cells were selected at each passage in culture, leading to selection of cells that formed closely packed colonies on plating. Experimental Lewis lung tumours were generated as described for the KHT tumours. MGH-U1 xenografts were generated by injecting $\sim 10^6$ cells, maintained in culture, into the left hind legs of immune-deprived CBA/CAJ mice. Mice were immune-suppressed by thymectomy, and treatment with cytosine arabinoside and whole body irradiation (Steel *et al.*, 1978; Kovnat *et al.*, 1982).

Animals bearing tumours were treated with *Pseudomonas 7A* glutaminase by i.p. injection, usually given as 4 injections at daily intervals. Controls received either saline or heat-inactivated glutaminase. Some groups of animals also received radiation to their tumours, delivered from a specially constructed double-headed 250 kVp X-ray machine at a dose rate of $\sim 11.4\,\mathrm{Gy\,min^{-1}}$. Animals irradiated in air were lead shielded during irradiation without anaesthesia, and the tumour-bearing leg was taped in the radiation field. Radiation was delivered at least 1 h after glutaminase injection on the third day of such injections; if glutaminase led to the death of nutrient-deprived and hypoxic cells this schedule should have allowed radiation effects to be directed against surviving aerobic cells. Some groups of animals received radiation to their KHT tumour under hypoxic conditions: this was achieved by applying a clamp to the tumour-bearing leg of anaesthetized mice for 5 min prior to and during radiation, as described previously (Tannock, 1982).

Response of the KHT tumour to treatment was assessed in coded animals by delay in tumour growth, as described previously (Tannock, 1982).

Response of the Lewis lung tumour and of MGH-U1 xenografts was assessed by clonogenic assay. Animals were killed and their tumours were removed 2 h after the last injection of glutaminase (about 24 h after radiation, in animals that received this treatment). The tumour was removed, chopped coarsely with scissors, and then stirred in 0.05% trypsin in calcium-free PBS for 30 min. This procedure led to a suspension of single cells with high viability and serial dilutions were plated in triplicate Petri dishes. Stained colonies were counted about 10 days later.

*Measurement of glutamine concentration*

The concentration of glutamine in medium or serum was determined by using a modification of the enzymatic method described by Pye *et al.* (1978). Pooled serum from mice was deproteinized using perchloric acid, which was added to give a final pH of 3.0; we found that glutamine was broken down if pH was allowed to fall to a lower value. The method is based on the conversion of glutamine to glutamic acid in the presence of glutaminase, followed by oxidation of glutamic acid in the presence of glutamate dehydrogenase, NAD, and ADP. The second reaction leads to the reduction of NAD to NADH which was measured with a fluorescence spectrometer (Perkin-Elmer model LS-3) using excitation and emission wavelengths of 350 nm and 460 nm respectively. This method allows the measurement of concentration of both glutamine and glutamic acid. Comparison of repeated estimates of glutamine and glutamic acid in known standard solutions indicated that the method was accurate to within $50\,\mu\mathrm{M}$ for each amino acid.

### Results

*Single cells in culture*

The growth of CHO and MGH-U1 cells in normal α-medium, in the absence of glutamine, and in the absence of both glutamine and glutamic acid is shown in Figure 1. CHO cells were able to grow in the absence of both amino acids, but their growth rate was suppressed; the plating efficiency of CHO cells taken from 5-day cultures without glutamine and glutamic acid was $<50\%$, as compared to 90% in controls. Human MGH-U1 cells grew more slowly under control conditions, and in duplicate experiments showed minimal or no growth in the absence of glutamine.

Cell survival was assessed following culture of CHO or MGH-U1 cells for up to 6 h in various types of deficient media. These experiments were designed to assess the acute effects of nutrient deprivation, as might occur in tumour cells that migrate toward a region of necrosis (Tannock, 1968). The results of a representative experiment are shown in Figure 2. Removal of glutamine with or without glutamic acid, or removal of glucose or oxygen alone had little effect on cell survival in multiple experiments, and differences in plating

**Figure 1** Growth curves for (A) CHO cells and (B) MGH-U1 human bladder cancer cells grown in α-medium plus 10% dialyzed FCS, or in similar medium lacking glutamine ± glutamic acid. Repeated experiments were qualitatively similar, although growth rate of the cells varied among experiments.

efficiency under these conditions were not significant. Medium deficient in glucose and glutamine (the major substrates for energy metabolism) led to some fall in cell survival over 6 h; this fall in survival was greater when cells were exposed to nitrogen, thus depriving cells of the ability to use the Krebs cycle. Under no conditions, however, did nutrient deprivation lead to rapid killing of cells, suggesting that many cells can maintain vital functions for several hours in the absence of substrates for energy metabolism.

Incubation of α-medium at 37°C with 0.01 or 0.1 IU ml$^{-1}$ glutaminase at pH 7.2 led to a fall in glutamine concentration to undetectable levels after ~8 h or within 2 h respectively (data not shown). The influence of glutaminase on cell survival was assessed following a 24 h exposure of both CHO and MGH-U1 cells. The enzyme was added to α-medium which either contained glutamine or was glutamine free (Figure 3). CHO cells had no change in plating efficiency after 24 h in glutamine-free medium. In the initial presence of glutamine (0.2 or 2 mM), glutaminase concentrations of 0.01 to 0.1 IU ml$^{-1}$ led to only a small fall in cell survival of CHO cells to the range of 70–90%, suggesting minimal direct toxicity. There was a greater reduction in survival (to ~20%) in glutamine-free medium. For MGH-U1 cells removal of glutamine alone for 24 h caused a fall in cell survival to about 30%, but additional toxicity of glutaminase was of the same order as for CHO cells (data not shown).

### *Spheroids*

MGH-U1 spheroids showed a linear increase in diameter of 80–100 μm per day when grown under control conditions (2 mM glutamine). Spheroids grew at the same rate as controls in glutamine concentrations above 0.1 mM (data not shown), but grew more slowly when the glutamine concentration was reduced to 0.1 mM or below (Figure 4). Spheroids did not grow in glutamine-free medium. There was also no growth of spheroids in α-medium containing 0.01 IU ml$^{-1}$ glutaminase, regardless of whether or not glutamine was present initially in the medium.

**Figure 2** Cell survival of MGH-U1 cells after culture for 0–6 h under various conditions of nutrient deprivation. Mean and range are indicated for triplicate plates.

**Figure 3** Cell survival of CHO cells following incubation for 24 h with varying concentrations of *Pseudomonas 7A* glutaminase. Medium either contained glutamine 2 mM (○ ●) or 0.2 mM (△) or was glutamine free, (▽ ▼) prior to addition of glutaminase. Open and closed symbols represent results of independent experiments.

**Figure 4** Growth of MGH-U1 spheroids in different concentrations of glutamine. Mean ± s.e. is indicated for groups of at least 6 spheroids.

Histological sections of spheroids that had grown in varying concentrations of glutamine (0, 0.05, 0.1 or 2 mM) for 6–7 days were examined for the presence of central necrosis. These spheroids had mean diameters in the range of 400–1000 μm, depending on the glutamine concentration in which they were grown, but there was no necrosis in any of them. This result may be contrasted to previous studies which have demonstrated extensive necrosis in MGH-U1 spheroids cultured in glucose-deficient medium (Tannock & Kopelyan, 1986*a*).

### *In vivo experiments*

The toxicity of glutaminase, given as daily injections for four days was studied in C3H and C57 mice. Maximum tolerated doses were about 150 IU $kg^{-1}$ $day^{-1}$ and 500 IU $kg^{-1}$ $day^{-1}$ respectively in tumour-bearing mice, although higher doses were tolerated by C3H mice without tumours. Mice bearing the KHT tumour showed an increase of serum lactate dehydrogenase (LDH) levels to ~20,000 IU $ml^{-1}$ as compared to a mean value of 2300 IU $ml^{-1}$ in controls; this suggests that the KHT tumour is infected with the LDH-elevating virus which has been shown by others to delay the clearance of glutaminase and to increase its toxicity (Riley *et al.*, 1974; Roberts *et al.*, 1979).

**Figure 5** Cell survival in (A) Lewis lung tumours and (B) MGH-U1 human bladder cancer xenografts as a function of radiation dose following treatment with radiation alone (closed symbols), or radiation plus glutaminase (500 IU kg$^{-1}$ i.p. $\times$ 4 days; open symbols). Different symbols represent independent experiments, and mean and range are indicated for triplicate plates. Both sets of data were fitted by single regression analyses, for doses >4 Gy, and the resulting cell survival curves are characterized by (A) $D_0 = 3.1$ Gy, $\bar{n} = 2.0$, and (B) $D_0 = 4.6$ Gy, $\bar{n} = 1.8$.

No increase in serum LDH was observed in C57 mice bearing the Lewis lung tumour, which presumably lacks the virus.

Mean glutamine levels in the serum of C3H mice were in the range of 0.3–0.4 mM. Serum glutamine levels at intervals after injection of 100 IU kg$^{-1}$ glutaminase into C3H mice (without tumours) fell rapidly to the range of 0–20% of concurrently determined control values within 2 h and remained at low levels for 24 h (data not shown).

Cell survival in Lewis lung tumours and MGH-U1 xenografts following radiation alone, and after radiation given on the third day of four glutaminase injections are shown in Figure 5. Glutaminase had no consistent effect on cell survival, either when used alone or with radiation; this result implies no effect of glutaminase (and glutamine depletion) on the hypoxic cells that survive radiation treatment in this tumour.

Glutaminase did cause a dose-dependent increase in growth delay of the KHT tumour. When used with 15 Gy radiation to the tumour, the increase in growth delay caused by glutaminase was equal or slightly greater to that caused by glutaminase alone (Figure 6). In order to determine whether nutrient-deprived and hypoxic cells that are spared by radiation might be killed by glutamine depletion, we administered glutaminase to animals that received radiation to their tumours under either aerobic or hypoxic conditions. Radiation delivered under hypoxic conditions would be expected to kill cells uniformly throughout the tumour and will not therefore select a surviving population of nutrient-deprived cells. There was a slightly larger effect of glutaminase when used with radiation delivered under aerobic as compared to hypoxic conditions (data not shown), but the interaction of the drug with either aerobic or hypoxic radiation was within the range of additivity.

### Discussion

We have studied the effects of glutamine

**Figure 6** Growth curves for the KHT tumour treated with (A) glutaminase alone, or (B) 15 Gy irradiation ± glutaminase. The radiation was given on the third day of glutaminase injections. Mean ± s.e. is indicated for groups of 7–8 tumours.

deprivation on single cells in culture, on spheroids, and on experimental tumours in order to provide models of increasing complexity which might detect an effect of glutamine depletion to interact with deficiencies of other nutrients to cause cell death. As reported by others (Eagle *et al.*, 1956) we found a cell line-dependent inhibition of growth under conditions of glutamine deprivation in tissue culture. Major effects to reduce cell viability during a 6 h incubation period were observed, however, only when glucose and oxygen were also deficient. These conditions are likely to suppress energy metabolism by preventing access to the Krebs cycle and oxidative phosphorylation, and by removing glucose and glutamine as sources of carbohydrate for glycolysis. We have shown previously that cell culture in the absence of glucose and oxygen also leads to loss of cell viability (although the effect is larger when glutamine is also absent), and to a fall in cellular ATP levels (Rotin *et al.*, 1986).

Low levels of glucose, oxygen, and glutamine might be expected to occur in the centre of multicellular tumour spheroids, due to limited penetration of these metabolites. We have shown previously that growth of MGH-U1 spheroids is reduced in medium deficient in glucose and/or oxygen (Tannock & Kopelyan, 1986*a*,*b*) in agreement with the results of others using different types of spheroids (Franko & Sutherland, 1979; Mueller-Klieser *et al.*, 1986). Central necrosis is also dependent on glucose and oxygen concentration in the medium surrounding spheroids. Spheroid growth was only influenced by glutamine concentration below 0.1 mM and spheroids did not develop necrosis when grown in either normal or glutamine-deficient medium. Thus, if spheroids are an appropriate model for nodules in tumours, it seems unlikely that glutamine levels are critical for maintenance of cell viability, since blood concentration is usually above 0.3 mM.

L-asparaginase is used clinically in the treatment of lymphoid malignancies, and asparaginase-glutaminase enzymes have been reported to exert therapeutic effects against several experimental tumours. These therapeutic effects were magnified by the presence of LDH-elevating virus which was found to delay the clearance of glutaminase (Riley *et al.*, 1974; Roberts *et al.*, 1979). C3H mice bearing the KHT tumour had elevated levels of serum LDH suggesting that this tumour was infected with the virus. There was no difference in serum LDH levels in C57 mice with or without Lewis lung tumours. The observation that glutaminase led to anti-tumour effects against the KHT, but not the Lewis lung, tumour may relate to the presence of the LDH-elevating virus, and its effect to delay the clearance of glutaminase. Even in mice without tumours, however, injection of glutaminase led to substantial reductions in the plasma level of glutamine for 24 h after injection.

If glutaminase exerts therapeutic effects through reduction of the serum concentration of glutamine, it might be expected that nutrient-deprived cells in tumours would be at high risk of cell death due to reduction of the low level of glutamine in their environment, and because of cumulative effects due to deficiency of other nutrients such as oxygen and glucose. To test this hypothesis, we studied the effects of glutaminase alone against murine solid tumours and also with radiation which was used to selectively deplete the aerobic, well-nourished cell population. Glutaminase, either alone or used with radiation, was without effect against Lewis lung tumours or MGH-U1 xenografts. The enzyme did cause dose-dependent growth delay of the KHT tumour, but the magnitude of its effects was not much greater when added to those of radiation, than when glutaminase was used alone. This result, and our experiments using the spheroid model, suggest that induction of glutamine deficiency is not likely to be very effective in causing the death of nutrient-deprived cells in solid tumours.

Supported by research grants CA 36913 and CA 40446 from the National Cancer Institute, NIH, USA, and by a grant from the National Cancer Institute of Canada.

### References

EAGLE, H., OYAMA, V.I., LEVY, M. & 2 others (1956). The growth response of mammalian cells in tissue culture to L-glutamine and L-glutamic acid. *J. Biol. Chem.*, **218**, 607.

ERLICHMAN, G. & TANNOCK, I.F. (1986). Growth and characterization of multicellular tumour spheroids of human bladder cancer origin. *In vitro.* (In press).

FRANKO, A.J. & SUTHERLAND, R.M. (1979). Oxygen diffusion distance and development of necrosis in multicell spheroids. *Radiat. Res.*, **79**, 439.

KOVNAT, A., ARMITAGE, M. & TANNOCK, I. (1982). Xenografts of human bladder cancer in immune-deprived mice. *Cancer Res.*, **42**, 3696.

McKEEHAN, W.L. (1982). Glycolysis, glutaminolysis and cell proliferation. *Cell Biol. Int. Rep.*, **6**, 635.

MOORE, J.V., HASLETON, P.S. & BUCKLEY, C.H. (1985). Tumour cords in 52 human bronchial and cervical squamous cell carcinomas: Inferences for their cellular kinetics and radiobiology. *Br. J. Cancer*, **51**, 407.

MUELLER-KLIESER, W., FREYER, J.P. & SUTHERLAND, R.M. (1986). Influence of glucose and oxygen supply conditions on the oxygenation of multicellular spheroids. *Br. J. Cancer.*, **53**, 345.

O'TOOLE, C.M., POVEY, S., HEPBURN, P. & FRANKS, L.M. (1983). Identity of some human bladder cancer cell lines. *Nature*, **301**, 429.

PINE, M.J. (1983). Depletions in glutamine, arginine, asparagine, isoleucine and ornithine pools of rodent and human tumors. *Proc. Amer. Assoc. Cancer Res.*, **24**, 47.

PYE, I.F., STONIER, C. & McGALE, E.H.F. (1978). Double-enzymatic assay for determination of glutamine and glutamic acid in cerebrospinal fluid and plasma. *Anal. Chem.*, **50**, 951.

RILEY, V., SPACKMAN, D., FITZMAURICE, M.A. & 3 others (1974). Therapeutic properties of a new glutaminase-asparaginase preparation and the influence of the lactate dehydrogenase-elevating virus. *Cancer Res.*, **34**, 429.

ROBERTS, J. (1976). Purification and properties of a highly potent antitumor glutaminase-asparaginase with antitumor activity. *J. Biol. Chem.*, **251**, 2119.

ROBERTS, J., SCHMID, F.A. & ROSENFELD, H.J. (1979). Biologic and antineoplastic effects of enzyme-mediated *in vivo* depletion of L-glutamine, L-tryptophan and L-histidine. *Cancer Treat. Rep.*, **63**, 1045.

ROTIN, D., ROBINSON, B. & TANNOCK, I.F. (1986). The influence of hypoxia and an acidic environment on the metabolism and viability of cultured cells: Potential implications for cell death in tumors. *Cancer Res.*, **46**, 2821.

SAUER, L.A., STAYMAN III, J.W. & DAUCHY, R.T. (1982). Amino acid, glucose and lactic acid utilization *in vivo* by rat tumors. *Cancer Res.*, **42**, 4090.

STEEL, G.G., COURTENAY, V.D. & ROSTOM, A.J. (1978). Improved immunosuppression techniques for xenografting of human tumours. *Br. J. Cancer.*, **37**, 244.

SEBOLT, J.S. & WEBER, G. (1984). Negative correlation of L-glutamine concentration with proliferation rate in rat hepatomas. *Life Sci.*, **34**, 301.

SUTHERLAND, R.M., McCREDIE, J.A. & INCH, W.R. (1971). Growth of multicell spheroids in tissue culture as a model of nodular carcinomas. *J. Natl Cancer Inst.*, **46**, 113.

TANNOCK, I.F. (1968). The relation between cell proliferation and the vascular system in a transplanted mouse mammary tumor. *Br. J. Cancer*, **22**, 258.

TANNOCK, I.F. (1970). Population kinetics of carcinoma cells, capillary endothelial cells, and fibroblasts in a transplanted mouse mammary tumor. *Cancer Res.*, **30**, 2470.

TANNOCK, I. (1982). Response of aerobic and hypoxic cells in a solid tumour to adriamycin and cyclophosphamide, and interaction of the drugs with radiation. *Cancer Res.*, **42**, 4921.

TANNOCK, I.F. & KOPELYAN, I. (1986*a*). The influence of glucose concentration on growth and formation of necrosis in spheroids derived from a human bladder cancer cell line. *Cancer Res.*, **46**, 3105.

TANNOCK, I.F. & KOPELYAN, I. (1986*b*). Variation of $pO_2$ in the growth medium of spheroids: Interaction with glucose to influence spheroid growth and necrosis. *Br. J. Cancer*, **53**, 823.

THOMLINSON, R.H. & GRAY, L.H. (1955). The histological structure of some human lung cancers and the possible implications for radiotherapy. *Br. J. Cancer*, **9**, 539.

WEBER, G. (1983). Biochemical strategy of cancer cells and the design of chemotherapy: GHA Clowes Memorial Lecture. *Cancer Res.*, **43**, 3466.

WINDMUELLER, H.G. & SPAETH, A.E. (1978). Identification of ketone bodies and glutamine as the major respiratory fuels *in vivo* for postabsorptive rat small intestine. *J. Biol. Chem.*, **253**. 69.